# Molecular Prevalence and Risk Factors of *Campylobacter* Infection in Puppies in the Nairobi Metropolitan Region, Kenya

**DOI:** 10.1155/2023/8813405

**Published:** 2023-04-08

**Authors:** Sharon N. Mbindyo, Jafred M. A. Kitaa, Tequiero O. Abuom, Gabriel O. Aboge, Daniel W. Muasya, Beatrice W. Muchira, Nduhiu Gitahi, Charles M. Mulei

**Affiliations:** ^1^Department of Clinical Studies, Faculty of Veterinary Medicine, University of Nairobi, P.O. Box 29053-00625, Kangemi, Kenya; ^2^Department of Public Health, Pharmacology and Toxicology, Faculty of Veterinary Medicine, University of Nairobi, P.O. Box 29053-00625, Kangemi, Kenya

## Abstract

*Campylobacter* species are widely distributed pathogens; however, data on its epidemiology in puppies remain scanty, especially in Kenya. A cross-sectional study was conducted in the Nairobi Metropolitan Region to determine molecular prevalence and associated risk factors of *Campylobacter* species infection in puppies. A total of 260 rectal swabs were collected from puppies from breeding kennels, shelters, and the University of Nairobi Veterinary Teaching and Referral Hospital. The samples were subjected to polymerase chain reaction (PCR) assays for identification of *Campylobacter* species. Data on potential risk factors associated with puppy exposure were collected using a semistructured questionnaire. Multivariable mixed effects logistic regression analyses were performed with kennels as random effects. *Campylobacter* species were detected in 64 of the 260 sampled puppies yielding an overall prevalence of 24.6%. Multivariable results showed that puppies from shelters, puppies from kennels that are washed daily, puppies with a recent history of vomiting, and those treated with antibiotics in the past month were significantly associated with the presence of *Campylobacter* species. Being a kenneled puppy and having had concurrent bacterial infections were identified as protective factors. This study provides molecular evidence of puppy exposure to *Campylobacter* species which could have impact on puppy health and highlights the need to develop awareness and management strategies to potentially reduce the risk of transmitting this pathogen among puppies, to humans, and other animals.

## 1. Introduction

Campylobacteriosis, caused by thermophilic bacteria of the genus *Campylobacter*, is a significant zoonotic gastrointestinal disease affecting humans and animals, including dogs, globally [1–4]. The vast majority of *Campylobacter* infections in humans are attributable to the consumption of contaminated or undercooked poultry [2, 5, 6], contaminated water [7], or raw milk [1]. Close contact with pets has also been identified as a significant source of human *Campylobacter* species infections [8, 9] with dogs, particularly puppies (less than one year), serving as potential reservoirs of *Campylobacter* infection for their owners, with infants and young children having a higher risk of infection [10].

*Campylobacter* species prevalence in dogs varies widely [11–15], depending on age, geographic region, housing, diagnostic method, clinical history (diarrheic versus non-diarrheic dogs), and the presence of infection or concomitant disease [8, 16, 17]. Feeding homemade and commercial diets, compost exposure, and outdoor water access have all been linked to *Campylobacter* colonization in dogs [18–20]. The infection has also been linked to purebred dogs, concurrent enteric disease, and antibiotic treatment [21, 22]. Furthermore, when compared to adult dogs, younger dogs are more likely to become infected with *Campylobacter* species [12].

Though the detection of *Campylobacter* species is generally performed using the conventional culture method, it is time-consuming and labor-intensive, due to the fastidious nature of the species [23]. Hence, molecular-based assays, like polymerase chain reaction (PCR) and sequencing enable rapid and precise detection [1, 24, 25].

Despite reports of puppies serving as essential reservoirs for *Campylobacter* pathogens, current data on *Campylobacter* species epidemiology in Kenyan puppies are limited. Therefore, this study aimed at determining the molecular prevalence and associated risk factors of *Campylobacter* species in puppies in the Nairobi Metropolitan Region, Kenya.

## 2. Materials and Methods

### 2.1. Ethical Approval

This study was approved by the Biosecurity, Animal Use, and Ethics Committee (BAUEC) of the Faculty of Veterinary Medicine, University of Nairobi, Kenya (FVM BAUEC/2019/237). Verbal consent was sought from breeders, kennel managers, and puppy owners prior to sampling.

### 2.2. Study Area and Design

**Figure 1:**
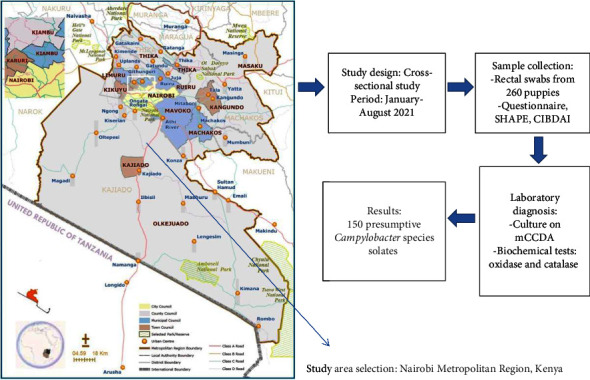
Schematic diagram of study design indicating the study area, puppy sampling, and laboratory diagnosis.

Study areas and design have been previously described [26] (Figure 1). In brief, this study was a cross-sectional study undertaken between January 2021 and August 2021 in breeding kennels, shelters, and the University of Nairobi Veterinary Teaching and Referral Hospital in the Nairobi Metropolitan Region, Kenya. These facilities were selected purposefully based on the high populations of puppies (less than one year) and the diversity of puppy breeds and management practices. Puppies in these facilities were randomly selected and sampled.

### 2.3. Sample Collection

The sampling methods have been previously described [26] (Figure 1). In brief, in order to determine potential risk factors associated with *Campylobacter* species infection, a detailed questionnaire was administered to collect puppy-level factors (age, breed, sex, vaccination status, and deworming status) and management factors (type of food, type of housing, kennel hygiene, and environmental hygiene). Prior to sampling, each puppy was assigned a body condition score (BCS) in accordance with the Waltham Size, Health, and Physical Examination (SHAPE) Score™ which contains seven scores from A (underweight) to G (obese) based on the presence and amount of subcutaneous and abdominal fat [27] (https://www.slideshare.net/WalthamCPN/waltham-pocket-book-of-healthy-weight-maintenance-for-cats-and-dogs-71137293). The Canine Inflammatory Bowel Disease Activity Index (CIBDAI) clinical scoring system by Jergens et al. [28] was used for the assessment of the puppies' general health status concerning gastrointestinal infection. The numerical index assesses the severity of illness based on the presence and frequency of six cardinal signs of gastrointestinal infection. Based on the total cumulative scores, the infection was classified as follows: clinically insignificant (0 to 3), mild (4 to 5), moderate (6 to 8), or severe (9 or greater). A total of 260 rectal swabs were then collected from the puppies: breeding kennels (*n* = 210), shelters (*n* = 6), and veterinary hospital (*n* = 44).

### 2.4. Polymerase Chain Reaction (PCR) Analysis for Identification of Genus *Campylobacter*

The isolates utilized in this study were obtained from a previous study on culture prevalence in puppies in Kenya [26] (Figure 1). *Campylobacter* isolates were isolated on mCCDA (Oxoid, CM0935) and identified by biochemical tests (oxidase and catalase tests). Genomic DNA was obtained from these presumptive *Campylobacter* species isolates using the boiling method as described by Wang et al. [29]. Polymerase chain reaction (PCR) amplifications were performed using a thermal cycler (Bio-Rad T100™ Thermal cycler). To confirm members of the genus *Campylobacter*, primers (C412GF 5′-GGATGACACTTTTCGGAGC-3′ and C1228R 5′- CATTGTAGCACGTGTGTC-3′) [30] targeting the *16S rRNA* gene were used. The polymerase chain reaction was performed in a total volume of 12.5 *μ*l containing mastermix of 6.25 *μ*l and 0.25 *μ*l each of forward and reverse primers, 5 *μ*l of DNA template, and 0.75 *μ*l of sterile distilled water.

The thermocycling conditions used were initial denaturation at 95°C for 15 minutes, followed by 25 cycles each of denaturation of 95°C for 30 seconds, annealing at 58°C for 1.5 minutes, extension at 72°C for 1 minute, and final heating at 72°C for 7 minutes. Samples were held at 4°C prior to analysis.

Controls were used for all PCR assays, and 10 *μ*l of amplified products was identified by electrophoresis in a 1.5% (weight/volume) agarose gel in 1X Tris-Borate-EDTA (TBE) buffer, subsequently stained with ethidium bromide and ran for 30–45 minutes at 200 V, and visualized by UV-illuminator (UVP GelMax 125 Imager, USA). The sizes of the amplicons were determined using 100 bp molecular ladder. Specific amplified fragments expected were of size 816 bp which corresponded to the *Campylobacter* genus.

The unit of observation corresponded to an individual sample, and each sample represented an individual puppy. If *Campylobacter* was detected by PCR in a sample, the puppy was considered infected.

### 2.5. Data Entry and Analysis

Questionnaire data and PCR results were entered into Microsoft Excel version 2016 (Redmond, WA, USA) before being exported to STATA 17.0 (StataCorp LLC, USA) for analysis. *Campylobacter* species prevalence and other demographic parameters were computed using descriptive statistics. The chi-square test was used to compare *Campylobacter* species carriage ratios between different categorical groups. Potential factors associated with *Campylobacter* species carriage in puppies were investigated using univariable logistic regression analysis. Covariates were retained in the model if statistically significant at  *p* ≤ 0.2 using a backward stepwise elimination procedure. All variables that showed an association with the outcome variable in the univariable logistic regression analysis (*p* < 0.05) were considered in the final mixed effects logistic regression analysis. Potential clustering of puppies within kennels was controlled by including kennels as a random effect in the modeling. Model fit was assessed by checking for multicollinearity, overall goodness of fit of the model, influential data points, and outliers.

## 3. Results

### 3.1. Molecular Prevalence of *Campylobacter* Species Infection in Puppies in the Nairobi Metropolitan Region, Kenya

**Figure 2:**
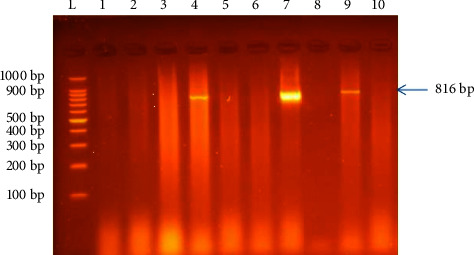
Representative PCR amplicons of *Campylobacter 16S rRNA* gene. Lane L: molecular ladder (100 bp); lanes 4, 7, and 9: positive samples showing amplicon at approximately 816 bp; lanes 1, 2, 3, 5, 6, and 8: no amplicons observed; lane 10: negative control.

Polymerase chain reaction was used to identify *Campylobacter* species isolates obtained from a previous study [26]. This was done by targeting the *16S rRNA* gene specific to *Campylobacter* species which produced a specific band corresponding to the expected size of 816 bp (Figure 2). The results from PCR analysis revealed a molecular prevalence of 24.6% (64/260).

### 3.2. Descriptive Statistics of Variables and Univariable Logistic Regression Analysis of Potential Risk Factors for Puppy *Campylobacter* Species Positivity (*p* ≤ 0.2)

The distribution of various puppy-level and management factors associated with *Campylobacter* species infections based on PCR is described in Table 1. A higher prevalence of *Campylobacter* species was observed in puppies from shelters (50% (3/6)) and those kept as pets (29.7% (11/37)). Similar observations were noted in puppies sharing a kennel (25.1% 55/219)), whose kennels were washed on a daily basis (27.1% (62/229)), from kennels with wooden floors (27.8% (49/176)), and puppies fed homemade diets (30.7% (27/88)).

Univariable logistic regression identified 11 factors to be associated (*p* **≤** 0.2) with positive *Campylobacter* species PCR status (Table 1). Four of the factors were associated with higher *Campylobacter* species carriage. They include kennels with concrete floors (OR: 2.3; *p* = 0.12), kennels that are washed daily (OR: 6.7; *p* = 0.0001), puppies with a history of recent vomiting (OR: 1.5; *p* = 0.2), and puppies treated with antibiotics in the past month (OR: 1.7; *p* = 0.093). Seven factors were associated with lower *Campylobacter* species carriage: puppies from breeding kennels (OR: 0.6; *p* = 0.003), puppies from shelters (OR: 3; *p* = 0.0001), puppies kept for security (OR: 0.3, *p* = 0.029), kenneled puppies (OR: 0.8, *p* = 0.005), puppies more than 5 months of age (OR: 0.3; *p* = 0.016), puppies with an ideal body condition or are moderately obese (OR: 0.6; *p* = 0.14), and puppies with concurrent bacterial infections (OR: 0.5, *p* = 0.2).

### 3.3. Multivariable Mixed Effects Logistic Regression Analysis of Significantly Associated Explanatory Variables for Puppy *Campylobacter* Species Positivity (*p* < 0.05)

Multivariable logistic regression analysis revealed that the factors significantly associated with higher *Campylobacter* species positivity at *p* < 0.05 were puppies from shelters (OR: 2.6, 95% CI: 1.9–3.6, *p* = 0.0001), kennels that are washed on a daily basis (OR: 11.4, 95% CI: 2.8–46, *p* = 0.001), puppies with a recent history of vomiting (OR: 3.4, 95% CI: 1.01–11.4, *p* = 0.046), and puppies treated with antibiotics in the past month (OR: 2, 95% CI: 1.11–3.6, *p* = 0.02). Protective factors identified were puppies from breeding kennels (OR: 0.65, 95% CI: 0.44–0.94, *p* = 0.024) and puppies with concurrent bacterial infections (OR: 0.18, 95% CI: 0.04 = 0.87, *p* = 0.033).

### 3.4. Prevalence of *Campylobacter* Species Infection Based on the Canine Inflammatory Bowel Disease Activity Index (CIBDAI) Clinical Scoring System

Fifty-four out of 260 puppies exhibited one or more of the six cardinal signs of gastrointestinal infection used to assess the degree of illness. Polymerase chain reaction identified *Campylobacter* species in 36.4% and 13.7% of the puppies whose infection status was classified as severe and clinically insignificant, respectively (Table 2).

## 4. Discussion

The study of zoonotic diseases such as campylobacteriosis is necessary due to the increasing number of people keeping dogs in their homes. Given that dogs, especially puppies, can be reservoirs of pathogenic *Campylobacter*, it is necessary to increase information about the epidemiology of this disease in these animals.

Polymerase chain reaction (PCR) detected 64 *Campylobacter* species (24.6%, 64/260) by targeting the *16S rRNA* gene specific for these microorganisms. This proportion was within the range of 8.58% to 75.7% reported in studies done in the past five years [15, 31–33]. The relatively high prevalence of thermophilic *Campylobacter* species observed in this study among puppies is a cause for concern, as their feces contaminate the environment and may serve as a source of infection for humans, particularly children.

Though clinical manifestations of gastroenteritis include diarrhea and vomiting [34], this study found no significant association between diarrhea occurrence and *Campylobacter*-positive status, a finding that is in agreement with previous research [35, 36]. However, this study found a statistically significant association between vomiting in puppies and the isolation of *Campylobacter* species. This finding contradicts those of Verma et al. [11], who found no correlation between *Campylobacter* species infection and the incidence of vomiting in dogs. Findings of this study, however, concur with those of Guest et al. [37], who found a link between gastrointestinal signs and *Campylobacter* species infection in puppies.

Shelter-housed puppies were at a higher risk for *Campylobacter* species carriage. These findings are in agreement with those of previous studies [36, 38]. *Campylobacter* species carriage is more prevalent among puppies who share a habitat with other puppies such as in shelters [12, 39, 40]. This may be due to the fact that puppies are from multiple sources and the stress of comingling predisposes them to stress and vices such as coprophagia which may lead to the ingestion of these bacteria, resulting in further infection, shedding in feces, and contamination of the environment [41]. The puppies may also roll in the feces, contaminating their fur [42] and further spreading the bacteria to surfaces with which they come into contact.

It is recognized that kennel hygiene is a potential risk factor for *Campylobacter* species carriage in dogs [43]. In this study, the daily washing of kennels was a highly significant risk factor for *Campylobacter* species carriage in the studied puppies. Despite the fact that daily washing of kennels improves hygiene, *Campylobacter* species are sensitive to desiccation and do not survive in dry environments [44], and thus daily washing increases their survivability in kennels as well as increases the chance of contamination of water sources [45], allowing water to be a vehicle for dissemination [46]. Puppies may also lick the residue water, resulting in pathogen ingestion.

Treatment with antibiotics in the past month was greatly significant with the risk of *Campylobacter* species carriage which was in agreement with a study by Leonard et al. [18]. This could be due to the inappropriate use of antibiotics in the treatment of other systemic infections with campylobacteriosis co-infection, thus promoting the emergence of antimicrobial resistant strains of *Campylobacter* species.

## 5. Conclusion

This study has shown that puppies in Kenya carry *Campylobacter* species which can be transmitted to humans and other animals through contaminated environmental sources. Polymerase chain reaction (PCR) should be regarded as indispensable in clinical and epidemiological research. It is important to develop awareness and management strategies to potentially reduce the risk of transmitting this pathogen from puppies to humans and other animals.

## Figures and Tables

**Table 1:** Descriptive statistics of variables and univariable logistic regression analysis of potential risk factors for puppy *Campylobacter*species positivity (*p* ≤ 0.2).

Variables	Level	Proportion (*n* = 260)	No. positive(%)	Odds ratio	*p* value
Type of facility	Veterinary hospital	44	13 (29.5)	Ref	
	Breeding kennels	210	48 (22.9)	0.6	0.003^∗^
	Shelters	6	3 (50)	3	0.0001^∗^

Reason for keeping puppy	Commercial	171	45 (26.3)	Ref	
	Pet	37	11 (29.7)	0.8	0.592
	Breeding	17	4 (23.5)	0.8	0.704
	Security	35	4 (1.4)	0.3	0.029^∗^

Type of housing	Household	11	0	Ref	
	Kenneled	249	64 (25.7)	0.8	0.005^∗^

Nature of housing	Individual	41	9 (22)	Ref	
	Grouped	219	55 (25.1)	1.2	0.73

Type of floor in the kennels	Wooden	176	49 (27.8)	Ref	
	Concrete	84	15 (17.9)	2.3	0.12^∗^

Daily washing of the kennels	Yes	229	62 (27.1)	6.7	0.0001^∗^
	No	31	2 (6.5)	Ref	

Type of food	Commercial	56	13 (23.2)	Ref	
	Homemade	88	27 (30.7)	0.79	1.2
	Others	116	24 (20.7)	0.65	0.76

Sex of the puppy	Male	123	23 (18.7)	Ref	
	Female	137	41 (30)	1.7	0.21

Age of the puppy	<2 months	90	28 (31.1)	Ref	
	2–5 months	108	27 (25)	0.6	0.29
	>5 months	62	9 (14.5)	0.3	0.016^∗^

Breed of the puppy	Local	52	15 (28.8)	Ref	
	GSD	96	29 (30.2)	0.9	0.78
	Others	112	20 (17.9)	0.5	0.24

Deworming status	Not up to date	105	26 (24.8)	Ref	
	Up to date	155	38 (24.5)	0.9	0.8

Vaccination status	Not up to date	63	16 (25.4)	Ref	
	Up to date	197	48 (24.4)	0.9	0.3

Body condition (SHAPE)	Thin and lean	117	34 (29)	Ref	
	Ideal and moderately obese	143	30 (21)	0.6	0.14^∗^

Recent diarrhea	Yes	36	9 (25)	1.2	0.71
	No	224	55 (24.6)	Ref	

Recent vomiting	Yes	14	5 (35.7)	1.5	0.2
	No	246	59 (24.6)	Ref	

Diagnosed with parvoviral enteritis	Yes	12	4 (33.3)	1	0.51
	No	248	6 (2.4)	Ref	

Diagnosed with helminthiasis	Yes	26	7 (27)	1.4	0.56
	No	234	57 (24)	Ref	

Concurrent bacterial infections	Yes	18	3 (16.7)	0.5	0.2^∗^
	No	242	61 (25.2)	Ref	

Recent treatment with antibiotics	Yes	42	14 (33.3)	1.7	0.093^∗^
	No	218	52 (23.9)	Ref	

Exposure to pets	Yes	90	18 (20)	0.8	0.7
	No	170	46 (27.1)	Ref	

Exposure to poultry	Yes	43	10 (23.3)	1.1	0.81
	No	217	54 (24.9)	Ref	

Exposure to livestock	Yes	16	3 (18.8)	0.7	0.63
	No	244	61 (25)	Ref	

^*∗*^Factors significant at *p* ≤ 0.2.

**Table 2:** Descriptive statistics of the occurrence of *Campylobacter* species infection in puppies based on the Canine Inflammatory Bowel Disease Activity Index (CIBDAI) clinical scoring system.

Infection status (CIBDAI)	No. tested	PCR positive (%)
Clinically insignificant	22	3 (17.4)
Mild	3	0
Moderate	18	9 (50)
Severe	11	4 (36.4)

## Data Availability

The data used to support the findings of this study are available from the corresponding author upon request.
